# Assembly and Activity of the WASH Molecular Machine: Distinctive Features at the Crossroads of the Actin and Microtubule Cytoskeletons

**DOI:** 10.3389/fcell.2021.658865

**Published:** 2021-04-01

**Authors:** Artem I. Fokin, Alexis M. Gautreau

**Affiliations:** ^1^Laboratoire de Biologie Structurale de la Cellule, CNRS, Ecole Polytechnique, IP Paris, Palaiseau, France; ^2^School of Biological and Medical Physics, Moscow Institute of Physics and Technology, Dolgoprudny, Russia

**Keywords:** HSBP1, FAM21, WASH, CCDC53, SWIP, strumpellin, dynactin, capping protein

## Abstract

The Arp2/3 complex generates branched actin networks at different locations of the cell. The WASH and WAVE Nucleation Promoting Factors (NPFs) activate the Arp2/3 complex at the surface of endosomes or at the cell cortex, respectively. In this review, we will discuss how these two NPFs are controlled within distinct, yet related, multiprotein complexes. These complexes are not spontaneously assembled around WASH and WAVE, but require cellular assembly factors. The centrosome, which nucleates microtubules and branched actin, appears to be a privileged site for WASH complex assembly. The actin and microtubule cytoskeletons are both responsible for endosome shape and membrane remodeling. Motors, such as dynein, pull endosomes and extend membrane tubules along microtubule tracks, whereas branched actin pushes onto the endosomal membrane. It was recently uncovered that WASH assembles a super complex with dynactin, the major dynein activator, where the Capping Protein (CP) is exchanged from dynactin to the WASH complex. This CP swap initiates the first actin filament that primes the autocatalytic nucleation of branched actin at the surface of endosomes. Possible coordination between pushing and pulling forces in the remodeling of endosomal membranes is discussed.

## Introduction

Active movement of cells or within cells is fueled by dynamics of cytoskeletal elements, such as actin filaments and microtubules together with associated molecular motors. Monomeric globular actin is polymerized into linear or branched structures of filamentous actin. Some linear actin cytoskeletons, e.g., stress fibers, can exert contractile forces by means of associated myosins, but branched actin structures also generate forces, pushing forces in this case, by their mere polymerization. The key role of branched actin is to remodel membranes during cell migration, endocytosis and intracellular trafficking.

Branched actin polymerization is due to a conserved and ubiquitous heptameric complex, the Arp2/3 complex, which nucleates a new actin filament off a pre-existing one (Pollard, 2007). The Arp2/3 complex is activated by a conformational change, in which the two Actin-related proteins it contains, Arp2 and Arp3, come into close proximity, thus mimicking the end of an actin filament, which can then initiate a new filament. Arp2/3 activity is promoted by the binding of Nucleation Promoting Factors (NPFs) to two sites on the Arp2/3 complex (Zimmet et al., 2020). Since an actin filament is required to generate new filaments by the Arp2/3 complex, the branching reaction is autocatalytic: the products of the reaction can become substrates of subsequent reactions. This raises the question of where the first filament comes from Achard et al. (2010). A number of answers have been proposed: short and freely diffusing actin filaments might be generated by cofilin-induced severing of previous filaments (Ichetovkin et al., 2002; Chen and Pollard, 2013), primer filaments might be nucleated by independent nucleators, such as formins or Spire (Zuchero et al., 2009; Isogai et al., 2015), or by the Arp2/3 complex itself activated in this case by atypical activators, such as SPIN90, which do not require a pre-existing filament (Wagner et al., 2013). These various mechanisms are not mutually exclusive and can be combined (Cao et al., 2020).

NPFs carry their Arp2/3 activation motif, commonly referred to as WCA, at their C-terminus. The WCA motif induces a conformational change of the Arp2/3 complex and loads a first actin molecule on the rearranged Arp2/3 (Pollard, 2007). WCA motifs are constitutively active, since they fold upon binding their partners, actin and Arp2/3 (Chereau et al., 2005; Derivery et al., 2009a; Zimmet et al., 2020). Therefore, in order to regulate Arp2/3 activation, WCA motifs must be masked either in an autoinhibited conformation, as for N-WASP, or within a stable multiprotein complex, as for WAVE (Derivery and Gautreau, 2010). Four families of NPFs, WAVE, WASH, WASP, and WHAMM, coexist in mammalian cells with an overall division of labor, consisting in activating the Arp2/3 complex at different subcellular locations (Molinie and Gautreau, 2018). For example, WAVE generates branched actin networks at the cell cortex and especially in adhesive protrusions such as lamellipodia, whereas WASH generates branched actin networks at the surface of endosomes and around centrosomes. Multiprotein complexes containing NPFs are responsible for the subcellular localization of the NPF, its maintenance in an inactive state and WCA exposure upon binding to upstream activators (Molinie and Gautreau, 2018).

## Assembly of Wash and Wave Complexes

### WASH and WAVE Are Regulated Within Analogous Complexes

WAVE has been purified from bovine brain and HeLa cells by classical chromatography and found to be contained within a pentameric complex (Sharon et al., 2002; Gautreau et al., 2004). A canonical WAVE complex is composed of CYFIP, NCKAP, ABI, WAVE and BRK1 subunits, with isoform variations due to the presence of 2 or 3 paralogous genes for 4 out of the 5 subunits. A detailed map of subunit interactions was derived from *in vitro* reconstitutions first and then crystallography (Gautreau et al., 2004; Innocenti et al., 2004; Chen et al., 2010). Overall a trimeric subcomplex composed of ABI-WAVE-BRK1 is covered by a platform made of a dimeric subcomplex composed of the large subunits NCKAP and CYFIP. The native and properly reconstituted WAVE complexes are inactive NPFs, because their WCA motif is masked (Derivery et al., 2009a; Ismail et al., 2009; Chen et al., 2010).

WASH has been purified by conventional and affinity chromatography and found to be also contained within a pentameric complex (Derivery et al., 2009b; Gomez and Billadeau, 2009; Jia et al., 2010). The WASH complex is composed of SWIP, Strumpellin, FAM21, WASH, and CCDC53 subunits. These subunits have been recently renamed WASHC1-5, but we will use here the original names for easier recognition in the vast majority of publications. A single gene usually encodes subunits of the WASH complex with the exception of WASH and FAM21, which are encoded by paralogous genes in mammalian genomes (Linardopoulou et al., 2007; Gomez and Billadeau, 2009).

Jia and colleagues have recognized analogous pairs of subunits in WAVE and WASH complexes using HHPred analysis (Jia et al., 2010). CYFIP corresponds to SWIP, NCKAP to Strumpellin, WAVE to WASH and BRK1 to CCDC53. The last subunit ABI also probably corresponds to FAM21, even though this pair is below the detection threshold. The HHPred analysis, which compares consensus sequences derived from multiple orthologs of the pair, catches distant relationships, which typically escape BlastP analysis. Indeed, no hybrid complex between WAVE and WASH subunits has been detected so far. In evolutionary distant species, when the WAVE gene is lost, the whole set of genes encoding WAVE complex subunits is also lost, and similarly for WASH (Veltman and Insall, 2010). These observations further highlight the importance of WAVE and WASH complexes and their distinct functions.

### Assembly Lines of WAVE and WASH Complexes

The question of how nascent subunits eventually assemble into a functional multiprotein complex has rarely been addressed, but has turned out to be important. In most cases, it appears that expression levels of subunits are somehow coordinated and that remaining excess subunits are degraded (Taggart et al., 2020). This is probably why multiprotein complexes are frequently destabilized when one of their subunits is missing in knock-down or knock-out experiments. This has been consistently reported for the WAVE complex (Kunda et al., 2003; Innocenti et al., 2005; Steffen et al., 2006; Derivery et al., 2008) and the WASH complex (Derivery et al., 2009b; Jia et al., 2010; Gomez et al., 2012; Visweshwaran et al., 2018).

**FIGURE 1:**
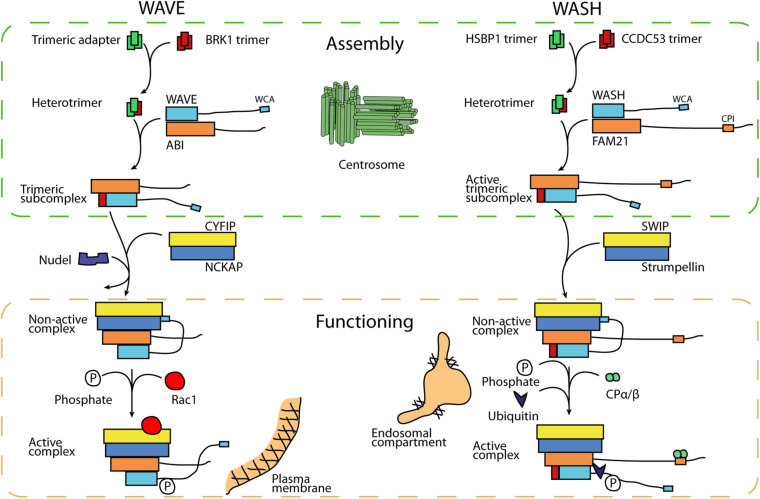
Model of assembly and activation of WAVE and WASH complexes. Smallest subunits, BRK1 and CCDC53 respectively, also exist as free trimers, which are precursors of the complexes. In order to assemble a first trimeric subcomplex, these trimers have to be dissociated in order to contribute a single subunit to complex assembly. HSBP1, a centrosomal protein, promotes this step in the case of the WASH complex. Nudel promotes the assembly of the pentameric WAVE complex. The WAVE complex is inactive and must be activated by the small GTPase Rac1 and through phosphorylation of the WAVE protein within the complex. The WASH complex, in contrast, is active already in its intermediate trimeric state. Activation of the fully assembled WASH complex requires phosphorylation and K63-linked ubiquitination of WASH.

The assembly of the WAVE complex was first recognized as a multi-step process. Its smallest subunit, BRK1, is the only subunit in excess in the cytosol (Gautreau et al., 2004) and this free form is a homotrimer assembled along a coiled coil (Derivery et al., 2008; Linkner et al., 2011). Within the WAVE complex, a single molecule of BRK1 associates with single molecules of ABI and WAVE through a heterotrimeric coiled coil (Chen et al., 2010). A dissociation step thus had to exist from the free homotrimer to the heterotrimer (Figure 1). But it is not known whether this transition is spontaneous or facilitated. In the analogous situation of the WASH complex, this process is facilitated.

The HSBP1 assembly factor promotes the homo- to hetero trimer transition of CCDC53, the BRK1 equivalent in the WASH complex. HSBP1, Heat Shock Factor Binding Protein 1, inhibits the HSF1 transcription factor, which is active as a trimer organized along coiled coils (Satyal et al., 1998; Pockley, 2003). HSBP1 itself is also a free homotrimer organized along a coiled coil (Visweshwaran et al., 2018). When the two homotrimers, HSBP1 and CCDC53, are mixed in the test tube, they spontaneously form a mixed heterotrimer, containing a single subunit of CCDC53 (Visweshwaran et al., 2018). So, this homo-to-hetero trimer transition, which was inferred for the WAVE complex, was shown to require an assembly factor in the case of the WASH complex.

The defect associated with HSBP1 inactivation in cells is the absence of a trimeric form of the WASH complex containing the 3 “small” subunits, CCDC53, WASH and FAM21 (but not Strumpellin, nor SWIP). An equivalent subcomplex was not detected in cells for the WAVE complex, but is likely to exist since the *in vitro* reconstitution of the WAVE complex involved combining the trimeric subcomplex of small subunits with the dimer of the large subunits NCKAP-CYFIP (Chen et al., 2010, 2014). Surprisingly, HSBP1 inactivation did not affect levels of the WASH pentameric complex, even though it did impair WASH associated functions in endosomal sorting (Visweshwaran et al., 2018). The WASH trimeric complex must thus be active. This conclusion is consistent with the fact that Strumpellin inactivation does not abolish WASH-dependent actin polymerization at the surface of endosomes (Tyrrell et al., 2016).

In the case of the WASH complex, it is not yet known how the pentameric complex is built. The intact levels of pentameric WASH in HSBP1 depleted cells argue against the simple addition of the dimeric complex to the trimeric complex, but this observation can also be accounted for if pentamers are built from trimers with a much faster rate than the one of trimer assembly. In the case of the WAVE complex, Nudel has been reported to promote pentameric WAVE assembly. Indeed this protein is engaged in multiple interactions with subunits of both WAVE subcomplexes (Wu et al., 2012). However, the structural transition in WAVE complex assembly that Nudel promotes is not yet established.

### The Centrosome, a Privileged Site for Complex Assembly

The assembly factor HSBP1 is very concentrated in the peri-centriolar material surrounding centrioles. This centrosomal localization has been seen in *Dictyostelium* ameba as well as in tissue and cell line of human origin (Visweshwaran et al., 2018). The other assembly factor, Nudel, is an adaptor of the dynein microtubule motor, which transports cargoes to the minus ends of microtubules. Consistently, Nudel accumulates at centrosomes (Feng et al., 2000). When cells are produced without centrosomes through the use of centrinone, a chemical inhibitor that blocks their duplication, HSBP1 becomes diffuse and the steady-state levels of WASH complexes are reduced (Visweshwaran et al., 2018). Together these observations suggest that the WASH complex, and probably the WAVE complex, are assembled at the centrosome. The concentration of reactants in a single defined location compared to the whole 3D volume of the cell might facilitate assembly of these multiprotein complexes.

The pericentriolar material is known to be composed of many coiled coil containing proteins (Kuhn et al., 2014), which could contribute to the homo-to-hetero trimer transition. The protein Pericentriolar Material 1 (PCM1) is a centrosomal coiled coil protein, which appears to anchor the WASH complex at the centrosome, where it promotes the nucleation of branched actin networks (Farina et al., 2016). WASH also binds to the centrosomal proteins, γ-tubulin and BLOS2 (Monfregola et al., 2010). The WASH complex is active at the centrosome (Farina et al., 2016). WASH-dependent centrosomal branched actin was found to anchor centrosomes to the nuclear envelope in interphase cells through the LINC complex (Obino et al., 2016). In mitotic cells, WASH-dependent centrosomal branched actin promotes the formation of spindle microtubules at the beginning of mitosis, in prometaphase (Plessner et al., 2019) and on the opposite might be responsible for the decrease of spindle microtubules during mitotic exit in anaphase (Farina et al., 2019).

Centrosomes are also a privileged site for the degradation mediated by the ubiquitin-proteasome pathway. Misfolded proteins are degraded by the ubiquitin-proteasome pathway and accumulate around centrosomes when degradation capacities are overwhelmed (Johnston et al., 1998; Kopito, 2000). The term aggresome has been coined to designate this central aggregation due to dynein mediated transport. Nudel was shown to participate in this transport of misfolded proteins (Wan et al., 2012). It actually makes sense that centrosomes are privileged sites for both assembly of multiprotein complexes and degradation of misfolded proteins. Indeed, complex assembly often fails for subunit unbalance: incomplete assemblies cannot reach the native state of the full complex, and thus degradation of improper assembly intermediates prevents their potential deleterious effects.

## Regulation of Wave and Wash Complexes

### Regulation of Levels and Activity

The regulation of levels of multiprotein complexes can in principle be achieved by the coordinated regulation of the expression of all subunits or by the regulation of the rate of their assembly, since subunits are only stable when part of their corresponding complex. There are numerous publications reporting up- or down- regulation of a single subunit from a multiprotein complex. For examples, the Arp2/3 and WAVE complexes are up-regulated in numerous cancers (references in Molinie and Gautreau, 2018), but the mechanisms responsible for these cases of deregulation are not known. The WASH complex is also up-regulated in mammary carcinomas (Visweshwaran et al., 2018). In this case, HSBP1 is also overexpressed, suggesting that tumor cells manage to produce more WASH complexes through the up-regulation of the HSBP1 assembly factor. Furthermore, HSBP1 overexpression is associated with poor metastasis-free survival of breast cancer patients.

Regulation of the activity of WAVE and WASH complexes is more often studied than the regulation of their levels. Activation of WAVE and WASH complexes involves the exposure of the so-called WCA motif that activates the Arp2/3 complex. At resting state, the WCA motif is masked and post-translational modifications of the N-terminal domains of WAVE and WASH subunits appear to play a critical role to release the masked WCA. Phosphorylation of Tyr 150 of WAVE2 by the Abl kinase is required for WAVE activity (Leng et al., 2005; Stuart et al., 2006; Chen et al., 2010). Similarly, phosphorylations of Tyr141 and 261 by Lck and Btk, respectively, were found to be critical for WASH activity (Huang et al., 2016; Tsarouhas et al., 2019). Polyubiquitination of WASH on Lys 220 with K63-linked chain synthesized by the MAGE-L2/TRIM27 E3 ubiquitin ligase activates the WASH complex in an *in vitro* assay (Hao et al., 2013).

The upstream regulation of WAVE and WASH complexes, however, considerably differ. The WAVE complexes depend on the small GTPase Rac1 for their recruitment and activation at the lamellipodial edge (Miki et al., 1998; Steffen et al., 2004). Rac1 collaborates with the other small GTPase Arf1 for WAVE activation (Koronakis et al., 2011; Humphreys et al., 2012). In contrast, among the many binding partners of the WASH complex, it is not clear which one is responsible for its activation, if any one of them is. Recruitment of the WASH complex at the surface of endosomes involves multiple binding sites for the retromer along the extended tail of the FAM21 subunit (Harbour et al., 2012; Jia et al., 2012; Helfer et al., 2013). However, endosomal recruitment of the WASH complex also occurs in the absence of functional retromer (McNally et al., 2017; Evans et al., 2020). HRS has been involved in retromer-independent recruitment, even if HRS and WASH do not belong to the same endosomal microdomains (MacDonald et al., 2018). Direct interaction of the WASH complex with endosomal lipids is also likely to contribute to endosomal recruitment (Derivery et al., 2009b, 2012). Phosphoinositides, such as phosphatidylinositol 3-phosphate (PI3P) and phosphatidylinositol 4-phosphate (PI4P), appear critical for WASH recruitment and activation on endosomal microdomains (Dong et al., 2016; Singla et al., 2019).

### Roles of the WASH Complex

The WASH complex is involved in endosomal cargo sorting. WASH was first implicated in the retrograde trafficking of the cation-independent mannose-6-phosphate receptor, CI-MPR, even if the retromer is not involved in CI-MPR trafficking against all expectations (Gomez and Billadeau, 2009; Kvainickas et al., 2017; Simonetti et al., 2017; Evans et al., 2020). WASH was repeatedly implicated in cargo recycling toward the plasma membrane, in the case of β2 adrenergic receptor (Puthenveedu et al., 2010), α5/β1 integrins (Zech et al., 2011), EGFR (Gomez et al., 2012), GLUT1 (Piotrowski et al., 2013; Lee et al., 2016), TCR (Piotrowski et al., 2013), ATP7A copper transporter (Phillips-Krawczak et al., 2015), LDLR (Bartuzi et al., 2016) and several others. This activity relies on interaction of the WASH complex with the so-called Commander assembly of complexes, composed of retromer and retromer-like components, retriever and CCC complexes (Phillips-Krawczak et al., 2015; Bartuzi et al., 2016; McNally et al., 2017; Chen et al., 2019).

The branched actin networks that WASH generates directly contribute to receptor sorting provided that they have an affinity for actin (Puthenveedu et al., 2010; MacDonald et al., 2018). Branched actin maintains lipidic microdomains diffusing in the endosomal membrane, because, when actin polymerization is impaired, they coalesce into a single domain, where WASH appears unable to detach from endosome membrane as seen by FRAP (Derivery et al., 2012). This behavior is best observed when endosomes are artificially enlarged by the expression of an active form of Rab5. Endosomes have occasionally been reported to clump when WASH is inactivated or actin polymerization is inhibited (Drengk et al., 2003; Derivery et al., 2009b; Gomez et al., 2012; Gautreau et al., 2014). This clumping phenotype might be related to the lack of dynamics of endosomal microdomains and the absence of their actin shell. But the most consistently reported phenotypes are pronounced tubulation of endosomes (Derivery et al., 2009b; Gomez and Billadeau, 2009), enlarged spherical endosomes (Gomez et al., 2012), or a mixture of both (Fokin et al., 2021). The reason for the predominance of one phenotype over the other is not known, but defective cargo sorting and defective generation of transport intermediates similarly characterize both phenotypes.

**FIGURE 2:**
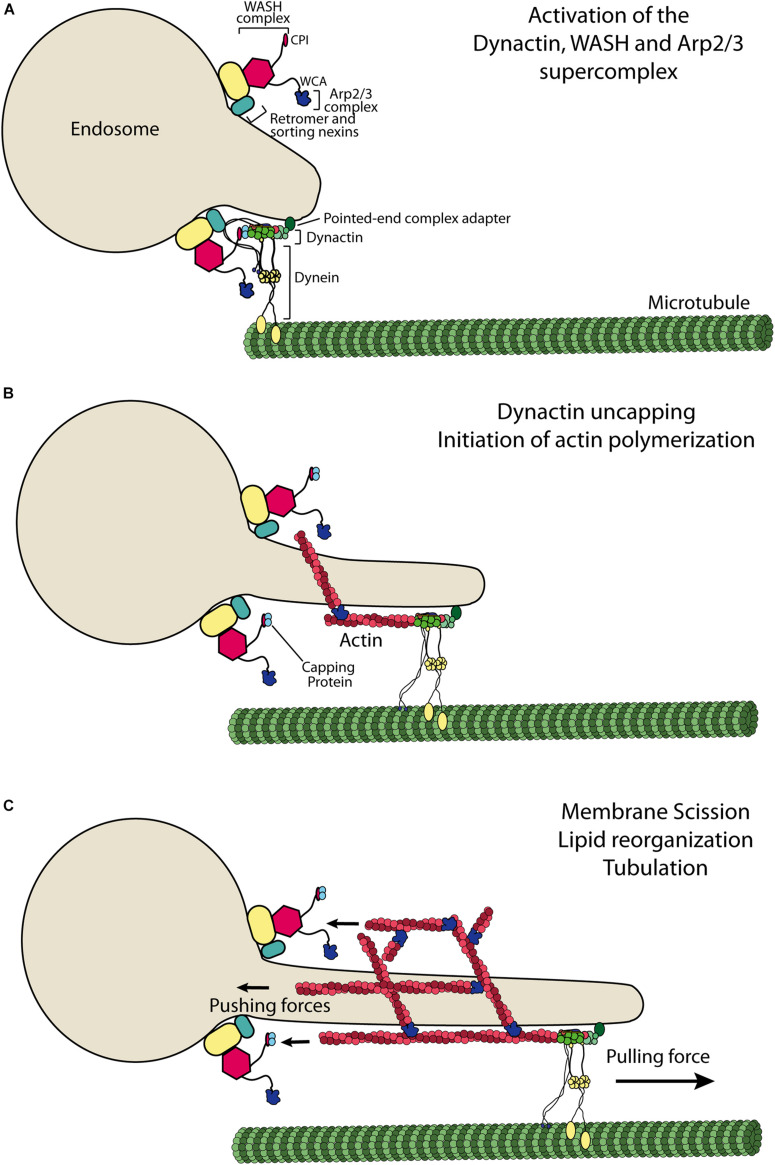
Model of WASH-dependent generation of endosomal branched actin. **(A)** Dynactin is an essential adaptor for dynein-mediated transport. Dynactin interacts with the WASH complex, which uncaps its actin-like minifilament through its CPI motif. **(B)** Uncapped dynactin elongates an actin filament, which can then serve as a substrate for Arp2/3 mediated branching induced by its WCA motif. **(C)** The actin-like minifilament of dynactin is embedded in the branched actin network and thus undergoes a retrograde flow. The membrane tube is elongated by the pulling force of dynein, whereas WASH-Arp2/3 at the neck of the membrane tube pushes against the bulk of the endosome. These opposing forces are likely to stretch the membrane and promote its scission. WASH dependent branched actin networks were also reported to regulate the organization of lipidic microdomains and endosomal tubulation.

Endosomes are tubulo-vesicular structures, so the tubulation phenotype in perturbed conditions is the exaggeration of a normal process. It simply reveals that pulling endosomal membranes by microtubule motors continues when branched actin formation is impaired. Tubular extensions containing sorted cargo proteins give rise to autonomous transport intermediates upon scission. When scission is blocked with a dynamin chemical inhibitor, WASH can be clearly localized at the base of endosomal tubules, at the right location to perform scission (Derivery et al., 2009b; Figure 2). When endosomes enlarge homogeneously, remaining spherical, upon WASH inactivation (Gomez et al., 2012), the defect can be interpreted as a role of branched actin in the generation of membrane tubules. Branched actin is required to stabilize tubules containing β2 adrenergic receptor (Puthenveedu et al., 2010). Mechanistically, branched actin would push against the bulk of the endosome in this case to contribute to tubule formation, in a manner most similar to the role of branched actin in yeast endocytosis (Kaksonen and Roux, 2018). In mammalian cells, branched actin surrounds the neck of clathrin-coated pits like a compressive collar and contribute to scission together with dynamin (Collins et al., 2011). Unfortunately, the ultrastructural organization of branched actin at the surface of endosomes is not yet elucidated. A major difference between endocytosis and the generation of transport intermediates from endosomes is that the latter require, in addition to branched actin, microtubules and microtubule motors, as seen in *in vitro* reconstitutions (Bananis et al., 2003; Murray et al., 2008).

### Role of Dynactin in Initiating Endosomal Branched Actin

Dynactin is a large megadalton complex that promotes the activity and processivity of the minus end directed microtubule motor dynein (Schroer, 2004). Dynactin is organized along a minifilament made of 8 molecules of Actin-related protein 1 (Arp1), one molecule of Arp11 and one molecule of β-actin (Urnavicius et al., 2015). This actin-like filament is capped by the capping protein. The capping protein, in fact a heterodimer of CPα and CPβ, terminates the elongation of actin filaments in the cytosol. The presence of an actin-like minifilament in a complex oriented toward microtubule and microtubule motors has remained a puzzle since its discovery almost 30 years ago (Lees-Miller et al., 1992). The WASH complex also recruits the capping protein. It does so through a so-called CPI motif at the C-terminus of the FAM21 subunit (Derivery et al., 2009b; Hernandez-Valladares et al., 2010; Jia et al., 2010). This CPI motif is unique to the WASH complex, as the equivalent subunit in the WAVE complex, ABI, rather displays an SH3 domain at its C-terminus.

Dynactin can be found throughout the cytosol, but it is enriched at the surface of endosomes (Habermann et al., 2001; Yeh et al., 2012), where it interacts with the coat complex ESCPE-1, composed of dimers of SNX1/SNX2 on the one hand and of SNX5/SNX6 on the other hand (Wassmer et al., 2009; Simonetti et al., 2019). Dynactin/dynein, as well as kinesins, participate in the tubulation of endosomes (Hunt et al., 2013; Brown et al., 2014; Delevoye et al., 2014; Marchesin et al., 2015). Dynactin can be colocalized and coimmunoprecipitated with the WASH complex, indicating that the two molecular machines directly or indirectly interact (Fokin et al., 2021). When mixed in the test tube, the CPI of the WASH complex appropriates capping protein from dynactin. The Arp1/11 minifilament of dynactin can then be elongated with actin and thus provides the first actin filament for the Arp2/3 branching reaction controlled by the WCA of the WASH subunit (Figure 2). This molecular scenario accounts for the facts that both dynactin and the CPI motif of FAM21 are required for the generation of endosomal branched actin networks.

The Arp1/11 minifilament of dynactin is embedded in the branched actin network it initiates. As a consequence, it should be subjected to the so-called retrograde flow due to extension of filaments abutting the WASH displaying membrane. It remains to be seen whether dynactin is complexed with dynein motors and adaptors when embedded in the branched actin network. It might well be, since the minifilament can accommodate dynein adaptors on its sides and elongate an actin filament from its barbed end. Structurally, there seems to be no steric hindrance (Urnavicius et al., 2015, 2018). If it is indeed the case, then dynein pulling the membrane tubule and branched actin pushing against the bulk of the endosomes stretch the neck of the tubule. This mechanical strain can favor either tubule elongation or scission of the tubule at its base, in agreement with the phenotypes described upon WASH knock-down or knock-out.

## Conclusion

Many studies of the WASH complex have been guided by its analogy with the WAVE complex. Its assembly bears a striking resemblance to the one of WAVE, as far as this homo-to-hetero trimer transition is concerned, for example. This allowed the identification of an assembly factor HSBP1, which reinforces the ties of WASH with the centrosome, that is a site of both assembly and functioning of the WASH complex. Studying the assembly of WASH also revealed a novel subcomplex, the trimeric WASH-CCDC53-FAM21 that appears to carry many, if not all, of the WASH associated functions. Assembly regulation seems to the major way that tumor cells use to upregulate the WASH complex in invasive cancers. The WASH complex is distinct, however, from the WAVE complex with the numerous interactions it makes with molecular machines associated with microtubules. This is naturally in line with the essential role of microtubules in the shape, motility and function of endosomes. One of the most striking examples of cooperation is the use of dynactin to generate a first primer filament and thus to initiate endosomal branched actin networks. Assembly of molecular machines and their collaboration within super-complexes are major challenges ahead of us for our understanding of the intimate functioning of WASH-dependent endosomal sorting. It requires endeavors in *in vitro* reconstitutions in combination with cell biology.

## Author Contributions

AF wrote a first draft of the manuscript and drew the figures. AG wrote additional parts of the text and homogenized the final manuscript. Both authors approved the final version of the manuscript.

## Conflict of Interest

The authors declare that the research was conducted in the absence of any commercial or financial relationships that could be construed as a potential conflict of interest.
